# Inverse Design of Energy‐Absorbing Metamaterials by Topology Optimization

**DOI:** 10.1002/advs.202204977

**Published:** 2022-12-11

**Authors:** Qingliang Zeng, Shengyu Duan, Zeang Zhao, Panding Wang, Hongshuai Lei

**Affiliations:** ^1^ Beijing Key Laboratory of Lightweight Multi‐functional Composite Materials and Structures Beijing Institute of Technology Beijing 100081 P. R. China

**Keywords:** energy‐absorbing structures, inverse design, metamaterials, passive pedestrian protection, topology optimization

## Abstract

Compared with the forward design method through the control of geometric parameters and material types, the inverse design method based on the target stress‐strain curve is helpful for the discovery of new structures. This study proposes an optimization strategy for mechanical metamaterials based on a genetic algorithm and establishes a topology optimization method for energy‐absorbing structures with the desired stress‐strain curves. A series of structural mutation algorithms and design‐domain‐independent mesh generation method are developed to improve the efficiency of finite element analysis and optimization iteration. The algorithm realizes the design of ideal energy‐absorbing structures, which are verified by additive manufacturing and experimental characterization. The error between the stress‐strain curve of the designed structure and the target curve is less than 5%, and the densification strain reaches 0.6. Furthermore, special attention is paid to passive pedestrian protection and occupant protection, and a reasonable solution is given through the design of a multiplatform energy‐absorbing structure. The proposed topology optimization framework provides a new solution path for the elastic‐plastic large deformation problem that is unable to be resolved by using classical gradient algorithms or genetic algorithms, and simplifies the design process of energy‐absorbing mechanical metamaterials.

## Graphical abstract

An inverse design method is proposed to realize the customization of energy‐absorbing metamaterials. The proposed algorithm designs a series of structures with ideal energy absorption curves and their performance is verified by the compression experiments. The functional energy‐absorbing structure is designed for car protection in different modes, demonstrating the feasibility and practicability of the proposed algorithm.

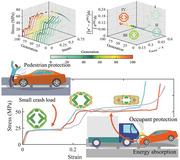

## 1 Introduction

Energy‐absorbing materials and structures are widely applied in aerospace, transportation, and human protection because of their excellent impact protection properties.^[^ ^1^ ^]^ Classical energy‐absorbing structures, such as columnar structures, sandwich structures, plate structures, honeycombs, and foams, have been thoroughly investigated in previous studies,^[^ ^2^ ^]^ whose performance can be tuned by changing geometric parameters. In order to further improve the energy absorption capacity, various energy absorption enhancement designs are proposed based on these classical structures. For example, aluminum with strong plasticity is selected as the material of the tube,^[^ ^3^ ^]^ composite materials are selected for material reinforcement,^[^ ^4^ ^]^ and grooves are added to the pipe walls for structural reinforcement.^[^ ^5^ ^]^ The improvement in energy absorption is realized by using the fold lines of the origami structure as plastic hinges,^[^ ^6^ ^]^ and the oblique surface of origami structure can also be selected to apply transverse shear energy absorption.^[^ ^7^ ^]^ Bio‐inspired design is another way to obtain structures with energy‐absorbing properties, by mimicking the internal structure of living matters.^[^ ^8^ ^]^ For example, concentric hexagonal energy‐absorbing panels are prepared based on the distribution characteristics of bones and bone marrow,^[^ ^9^ ^]^ and cross‐layered panels are prepared according to the microstructure of abalone shells.^[^ ^10^ ^]^ These designs are realized based on empirical or bio‐inspired configurations to achieve different energy absorption effects by adjusting geometric parameters and material types, which can be collectively referred to as the forward design method for energy‐absorbing structures. Specific energy absorption (SEA) and specific energy absorption per unit volume (SEA*_*ν*_*) are generally used to evaluate the energy absorption performance of these structures.^[^ ^11^ ^]^ Low initial peak crushing stress (IPCS), stable platform stress (*σ_m_*), and high densification strain (*ε_m_*) are the most important indicators in energy‐absorbing protection, and the shape of load‐displacement (stress‐strain) curve of an ideal energy‐absorbing structure should be rectangular or trapezoidal to ensure the efficient energy absorption effect.^[^ ^12^ ^]^ If the required energy absorption curve is set as a design goal to inversely design the structure and the base material is determined, then, the only design variable is the distribution of material. The inverse design method based on the topology optimization algorithm directly makes the structure meet the design goals without strict requirements on materials, simplifying the design process and improving the design efficiency.

The existing topology optimization algorithms are divided into two categories: gradient algorithms and intelligent algorithms. The gradient algorithms include the solid isotropic material with penalization method, the level set method, the phase field method, and the moving morphable components method.^[^ ^13^ ^]^ These methods require strict sensitivity derivation and exhibit a rapid convergence speed for solving independent variables based on finite element analysis. Intelligent algorithms include genetic algorithms and machine learning algorithms.^[^ ^14^ ^]^ Unlike gradient algorithms, these algorithms possess probability‐oriented numerical search and data features, leading to significant advantages in multiobjective optimizations. First, the topology optimization of energy‐absorbing structures needs to consider the large geometric deformation of the structure and the nonlinear material constitutive relationship. The existing metamaterial topology optimization studies, aiming at minimum compliance,^[^ ^15^ ^]^ negative Poisson's ratio,^[^ ^16^ ^]^ and multiscale optimization,^[^ ^17^ ^]^ are generally carried out under the assumption of small deformation and linear elasticity, rendering significant deviations under large deformations. Sigmund's group considered the finite deformation gradient,^[^ ^18^ ^]^ and realized the optimization of nonlinear negative Poisson's ratio structure up to 30% strain^[^ ^19^ ^]^ and nonlinear multimaterial structures.^[^ ^20^ ^]^ Though several constitutive models have been proposed in recent years,^[^ ^21^ ^]^ the methods considering both geometric nonlinearity and complex material constitutive models (elastoplastic and damage models) need to be established. Secondly, the gradient optimization algorithms require elemental representation of material‐free regions in the design domain to update design variables, resulting in mesh distortion under large deformations. Though new interpolation functions are proposed^[^ ^22^ ^]^ to resolve this issue, it is still difficult to implement the finite element analysis of complex structures for severe distortion, limiting the development of this type of algorithm in optimizing energy‐absorbing structures. Moreover, if the optimization design is based on the energy absorption curve, the sensitivity of gradient algorithm stems from the interpolation of structural deformation under different strains, which is difficult to provide an accurate optimization direction. Therefore, for the elastic‐plastic energy absorption problem under large deformation, the current gradient‐based topological optimization method faces an insurmountable bottleneck.

The genetic algorithm is a kind of intelligent algorithm, with the ability to solve problems such as minimum compliance optimization,^[^ ^23^ ^]^ independent mode control optimization,^[^ ^24^ ^]^ heat transfer optimization,^[^ ^25^ ^]^ and piezoelectric structure topology optimization.^[^ ^26^ ^]^ The gradient algorithm is difficult to solve the problems of complex multiobjective, variable history‐dependent or time‐dependent optimization. The genetic algorithm performs well in these problems, and its highly flexible model description brings better convergence. The most important point is that the genes of genetic algorithm do not need to describe the material‐free region,^[^ ^27^ ^]^ avoiding the grid problem. However, the optimization efficiency of genetic algorithm is lower than that of gradient algorithm due to the lack of sensitivity guidance, and the problem is more prominent when the structure undergoes large deformation. As a result, most existing research adopting the genetic algorithm concentrates on stiffness optimizations under small deformation.

**Figure 1:**
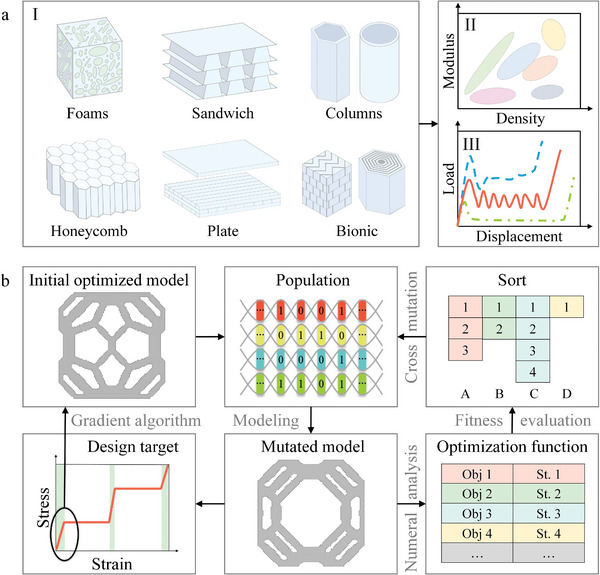
The forward and inverse design methods for energy absorbing structures. a) Traditional energy‐absorbing structures and design processes, where (I) shows the empirically or biologically inspired structure in the forward design, (II) represents a material database, and (III) shows the load‐displacement curves based on the forward design. b) Inverse design process of energy‐absorbing structures. The initial optimized model with different moduli is obtained by gradient algorithm and selected as the initial design population to meet the target curve under small deformation. After the population gene is processed, it is converted into a geometric model for numerical analysis. The algorithm calculates individual fitness according to the objective function and constraint function, and the sorted population undergoes genetic variation to form a new population. The structure satisfying the objective curve under large deformation is obtained through the iteration of optimization.

Different from the forward design method in **Figure** **1**a, which selects materials based on the structure and verifies the energy absorption curve, we propose an inverse design idea to generate structures from the target curve (Figure 1b). Herein, we carry out the topology optimization of multiobjective energy‐absorbing structures based on the nondominated sorting genetic algorithm II (NSGAII),^[^ ^28^ ^]^ and propose a series of structural mutation algorithms and design‐domain‐independent finite element mesh generation method to improve the efficiency of finite element analysis and optimization iteration. The initial optimized model with different moduli is obtained by gradient algorithm and selected as the initial design population to meet the target curve under small deformation. The structure satisfying the objective curve under large deformation is obtained through the iteration of optimization proposed in this paper. We have realized two types of designs: Design 1 is the structure with ideal energy‐absorbing curve that satisfies the target *IPCS*, stable *σ_m_*, and *ε_m_* >50%, and Design 2 is the energy‐absorbing structure that stabilizes *σ_m_* with multiplatforms. The material type is determined, the target energy absorption curve is proposed and an optimal structure is designed through the loop iteration of the optimization algorithm. As a first design example, we realize high energy‐absorbing structures with arbitrary *σ_m_*. In the second example, we demonstrate the design of a functional energy‐absorbing structure and propose a scheme of applying the structure to the energy‐absorbing protection of automobiles. Both designs are verified by additive manufacturing and experimental characterization. The proposed topology optimization framework provides a new solution path for the elastic‐plastic large deformation problem.

## 2 Result and Discussion

### 2.1 Optimization Algorithm and Convergence Verification

**Figure 2:**
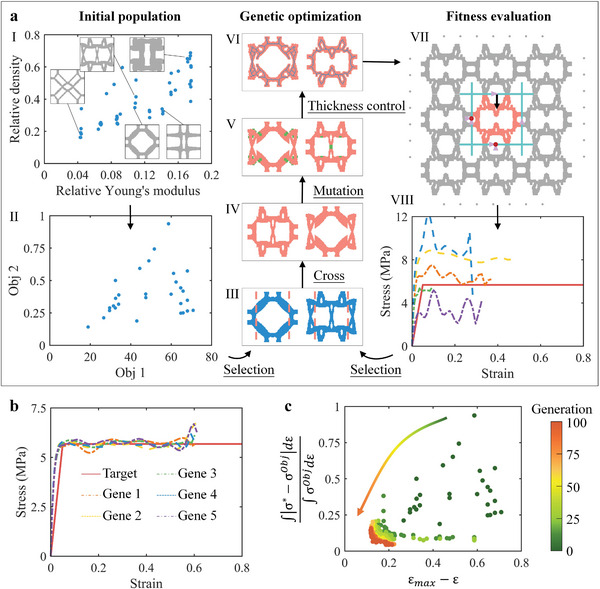
Topology optimization process and convergence verification of energy‐absorbing structures. a) I is the initial population, where the blue dots represent the relative density and equivalent property, and the gray image represents the corresponding structures. II is the objective function scatterplot, where the blue dots represent the function values calculated from the initial design. III shows the randomly selected parents, where the pink dotted line refers to the selected cross position. IV shows the children produced by III. V is the structure obtained by the mutation based on IV, where the green area indicates the mutation location. VI is the final progeny structure, where the blue line represents the centerline of the structure, and the pixel dilation algorithm and the skeleton algorithm are adopted to realize thickness control. VII represents the periodic boundary conditions during the finite element analysis. VII shows the load‐displacement curve calculated by the structure of the first generation, and the individual fitness is calculated from the curve as the basis to sort for the next generation. b) Comparison of stress‐strain curves of the optimal five‐gene individuals in the 100^th^ generation with the design target, where the solid line is the target curve. c) The convergence process of population objective function during the optimization process.

Structures with different moduli and densities were obtained based on stiffness optimization (Equation S2, Supporting Information), as the initial population shown in **Figure** **2**aI,^[^ ^29^ ^]^ to assist the algorithm rather than aimless search. The distribution of initial population in the objective function space was obtained through finite element analysis (Figure 2aII), and the probability of good genes in the offspring was improved through the optimization algorithm to perform nondominated sorting according to the individual objective function. Moreover, genetic strategies were proposed to assist more efficient crossover (Figure 2aIII–VI), mutation and thickness constraints to generate offspring genes. The genes of the offspring individuals were transformed into geometric models and periodic boundary conditions were imposed to perform compression analysis up to 80% strain to estimate the fitness (Figure 2aVII,VIII). The periodic boundary condition of the structure under large deformation was realized by adding four rigid plates, and the motion of plate and Master Node was coupled. The new population was generated by sorting the evaluation results of the offspring and parent together, and the algorithm loops were continued to obtain the results to meet the design requirements (Section S1.3, Supporting Information). A genetic strategy for metamaterials is developed (Figure S1–S4, Supporting Information), and the mesh problem in large deformations of the structure is overcomed (Figure S5d–f, Supporting Information). The thickness control of the structure is realized (Figure S5a–c, Supporting Information), and the efficiency of parallel computing is greatly improved (Figure S6, Supporting Information).

As a first example, 304 stainless steel was selected as the base material and the target *σ_m_* was set at 5.7 MPa. The small platform stress corresponds to a small equivalent density of the structure, and the stress‐strain curve is chosen so that the structure is more sensitive to material distribution. The target *ε_m_* was set to 0.8 to explore the limit of densification strain of the structure optimized by the algorithm. The objective function 1 was set to*ε*_max –_ *ε*, and objective function 2 was set to $\left(\int \right|\sigma^{*}-\sigma^{\mathrm{obj}}\left|d\varepsilon \right)/\left(\int \sigma^{\mathrm{obj}}d\varepsilon \right)$. Figure 2b shows the stress‐strain curves of the top five‐gene individuals after the 100^th^ generation of optimization. The gene individuals were remarkably close to the target curve with an average error of 5.28% and a maximum *ε_m_* of 0.6153. The distribution of individual objective functions of each generation in the solution space is shown in Figure 2c. With the increase of generation, the objective function gradually converges to the Pareto frontier, which proves that this algorithm possesses good convergence.

Small changes in the target curve affect the final configuration. Structures with different target curves for the same plateau stress are designed, as shown in Figure S9, Supporting Information. The target *σ_m_* was set to be 100 MPa and the detailed deformation process is shown in Figure S10, Supporting Information. The algorithm designs different configurations according to different target curves even if the target platform stress is the same. The design error of each configuration is less than 18% and *ε_m_* is greater than 0.57 (Table S2, Supporting Information), further confirming the diversity of the as‐designed configurations.

### 2.2 Inverse Design of Ideal Energy‐Absorbing Structures with Different Platform Stresses

**Figure 3:**
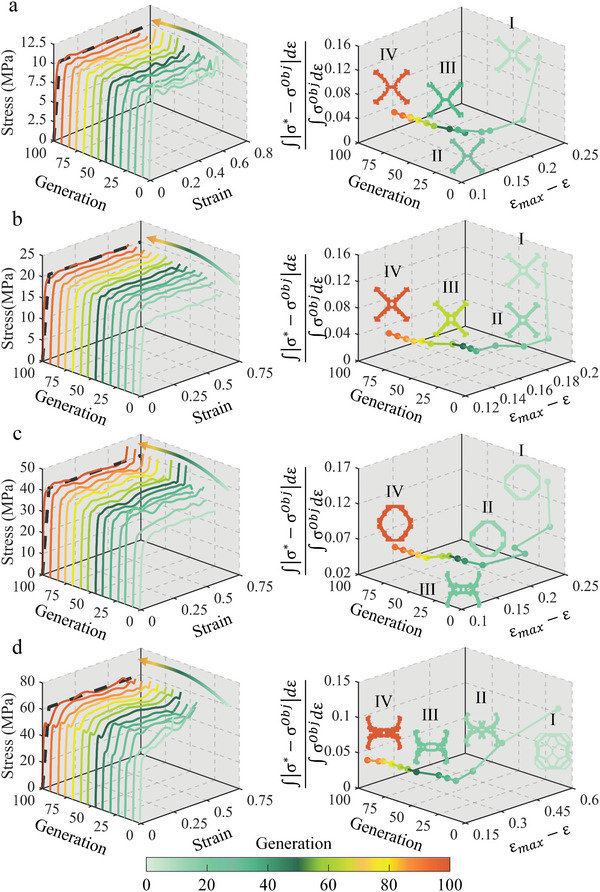
The optimization process for different *σ_m_* of a) 10 MPa, b) 20 MPa, c) 40 MPa, and d) 60 MPa. The optimal structure of each generation is represented by different colored solid lines and the black‐colored dotted line represents the target curve. The objective function values of the optimal structures of each generation are represented by different colored dots in the scattered plot, and straight lines connect the dots to show the optimization path. Four typical structures were selected and the structure color is consistent with the scatter color.

Herein, the design ability of the algorithm is further explored by setting the target *σ_m_* to 10 MPa, 20 MPa, 40 MPa, and 60 MPa as the optimization goal. The target *ε_m_* was set to 0.8 and the yield strain was set to 0.05. The target curve of each example is shown by the black‐colored dotted line in **Figure** **3**.

As shown in Figure 3a, the steepness of the stress‐strain curve of the optimal structure in the first few generations is quite different from the target curve. With the increase in number of generations, the curve gradually becomes smooth and converges to the optimal configuration after the 79th generation. It can be seen from the path of optimal configuration of the objective function space that the algorithm initially makes the structure approaching the target platform stress, gradually increases the *σ_m_*, and finally obtains the optimal solution. The structure of the first generation is a crossed rod with holes, as shown in Figure 3aI. In the 8th generation (Figure 3aII), the hole is optimized into a short connecting rod to reduce the *IPCS*. Finally, the rod gradually bends to smooth the stress‐strain curve and to increase the *ε_m_*. After the 100th generation of optimization, the optimal structure with stress‐strain curve error of 2.80% and *ε_m_* of 0.6628 is finally obtained.

Figure 3b shows the optimization results in the case of *σ* *_m_* = 20 MPa. Although the optimal configuration of the first generation is only slightly different from the previous example (Figure 3bI), the IPCS of this configuration obviously does not meet the requirements. With the increase of optimization generation, the hole in the middle of the configuration gradually becomes smaller and transforms from a rectangular shape to a circular shape, and the rod is gradually thickened to increase the load bearing capacity (Figure 3bII–IV). The stress‐strain curve of the optimal configuration gradually rises to the target. After the 100th optimization generation, the optimal structure is obtained with the stress‐strain curve error of 2.71% and *ε_m_* of 0.6622.

Figure 3c shows the case of *σ_m_* = 40 MPa. The optimal configuration is gradually optimized from the first generation of diamond‐shaped rods to the 8th generation of the top platform (Figure 3cI,II), and the shape of left and right connections is also gradually changing. A new deformation mechanism suddenly appeared in the 16^th^ generation (Figure 3cIII) and was surpassed in the 23rd generation. After the 100th optimization generation, the optimal structure became a ring structure with variable thickness rod connections. The error of the stress‐strain curve of the structure is 4.02% with a densification strain of 0.6590.

Compared with the first three examples, the case of *σ_m_* = 60 MPa in Figure 3d does not find a good configuration in the 1^st^ generation (Figure 3dI). From the stress‐strain curve, the IPCS of this configuration does not meet the requirements and there is no platform. The algorithm produces a structure (Figure 3dII) satisfies the original requirements through a continuous crossover variation at the 16th generation. The angle and thickness of rods are continuously optimized and the intermediate connection part is gradually thickened. After the 100th optimization generation, a structure dominated by rod bending is selected as the optimal configuration. The error of the stress‐strain curve of the structure is 4.34% and the *ε_m_* is 0.6630.

For the four cases with different platform stresses, the algorithm designs the structures with the stress‐strain error of less than 5% and *ε_m_* of greater than 0.6. In addition, Figure S12a,b, Supporting Information also shows the optimization process of examples with the target *σ_m_* of 140 MPa and 250 MPa, and Figure S13, Supporting Information shows the deformation process of the corresponding optimal structure. The equivalent modulus of the *σ_m_* = 250 MPa has reached 5% of the base material, confirming that the algorithm can design an energy‐absorbing structure with potentially any platform stress within a certain range.

### 2.3 Experimental Verification of an Ideal Energy‐Absorbing Structure

In this section, we verify the accuracy of inversely‐designed structures through experiments. The 5 × 3 lattice structure was designed to imitate the periodicity of structure in the computational model, and the experimental specimens were manufactured through laser powder bed fusion (L‐PBF). The compression experiments were carried out to obtain the stress‐strain curves of the structures, and the digital image correlation (DIC) technique was used to accurately measure the strain and deformation process.

**Figure 4:**
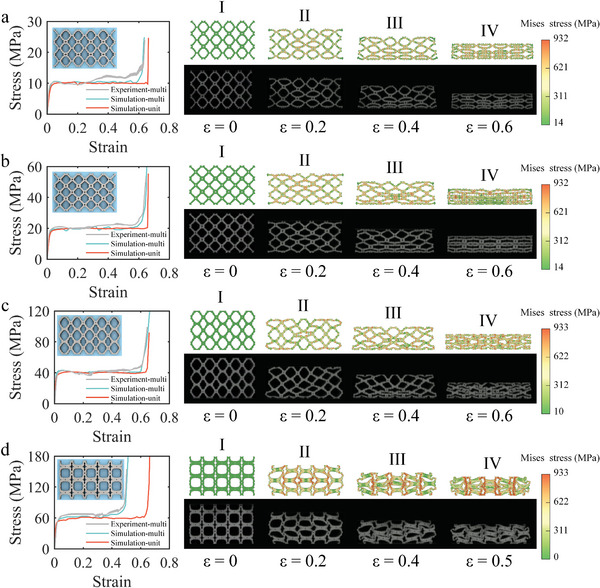
The compression experiment verification of energy absorbing structure. In the stress‐strain curve, the lattice experimental values, lattice simulation values, and unit cell simulation values are compared. The deformation process is compared on the right (I‐IV). a) *σ_m_* = 10 MPa. b) *σ_m_* = 20 MPa. c) *σ_m_* = 40 MPa. d) *σ_m_* = 60 MPa.

**Figure** **4**a shows the comparison between the experimental and simulation results of the case with *σ_m_* = 10 MPa. The stress‐strain curves of the structure are shown in the left figure, where experimental results are plotted as gray curves with continuous and ranged error bars. With the increase of strain, the cellular rods around the lattice started to bend gradually (Figure 4aI–III) and, finally, were densified at a strain of 0.6 (Figure 4aIV). The deformation of intermediate cells of the lattice is consistent with the deformation pattern of the unit cell under periodic conditions (Figure S11a, Supporting Information), which also proves that the error of the stress‐strain curve will be further reduced if the number of lattice cells is large enough. The experimental error of the lattice structure is 18.06% (Table S3, Supporting Information).

Figure 4b compares the experimental and simulation results of the 20 MPa example. The structure absorbs energy through rod nodes and the appearance of shear band does not affect the overall deformation model of the structure (Figure 4bII,III). Also, the final structure is smoothly compressed into a regular shape (Figure 4bIV). The experimental error of the lattice structure is 13.84%.

Figure 4c compares the experimental and simulation results of the 40 MPa example. The structure exhibits longer connecting sections and shorter rods, resulting in the utilization of nodal energy absorption before the strain of 0.4 (Figure 4cIII). Also, the bent rods absorb further energy after a strain of 0.4 (Figure 4cIV). The experimental error of the lattice structure is found to be 12.80%.

Figure 4d compares the experimental and simulation results of the 60 MPa example, where the structure directly absorbs energy through the bending of rods (Figure 4dII–IV). Unfortunately, the shear bands appearing in the structure lead to overall instability at the strain of 0.4. The connecting rods are stacked together with cell tilting, which causes the structure to enter the densification region. The experimental error of the lattice structure is found to be 19.11%. Although experimental conditions, e.g., the dimension of testing machine, limited number of unit cells affect the experimental results, the deformation mode and stress‐strain curve of the structure are still in good agreement with the designs in Section 2.2 (Figure S11d, Supporting Information). In addition, the thickness constraints of the algorithm facilitate the direct additive manufacturing of these designs (Figure S5c, Supporting Information).

### 2.4 Inverse Design of Multiplatform Energy‐Absorbing Structure for Automotive Protection

**Figure 5:**
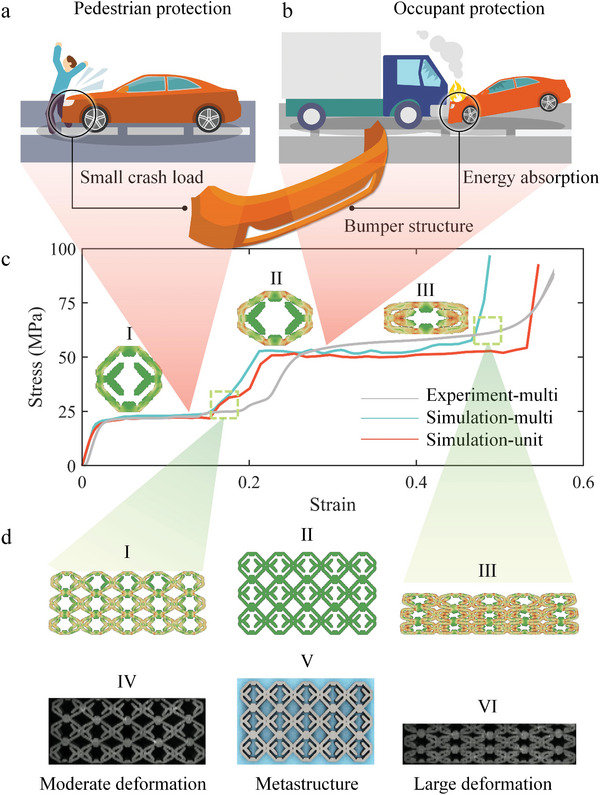
Inverse design of multiplatform energy‐absorbing structures. a) Pedestrian protection requires the structure to possess stable energy absorption characteristics for small crash loads. b) Occupant protection requires structures with high platform stress and energy absorption. c) Stress‐strain curves of lattice simulations, lattice experiments, and unit cell simulations of optimized dual‐platform structures, where I, II, and III represent the deformation states of the structure under different loads, respectively. d) Comparison of simulation and experimental results under different energy absorption modes, where I and IV are the deformation states of the lattice structure under small impact load, and III and VI are the deformation states of the lattice structure under high plateau stress.

In general, the energy‐absorbing structure is designed according to the service load, and an ideal energy‐absorbing stress‐strain curve exhibits a rectangular shape. In practical applications, the structure might need to have multiple platform segments to meet the energy absorption requirements under complex loads. The design of the total amount of energy absorption and trigger mechanism also requires that the stress‐strain curve must be accurately designed. For instance, in terms of vehicle energy absorption protection, when a car collides with a pedestrian, it is necessary to trigger the energy absorption mechanism with a small impact load to passively protect the safety of pedestrians, as shown in **Figure** **5**a. However, when a collision occurs between cars, the kinetic energy from the strong impact requires structural absorption with high platform stress (Figure 5b).

Herein, a multiplatform energy‐absorbing structure is proposed to solve this problem. *σ_m_* = 20 MPa and *σ_m_* = 50 MPa are set as the trigger stress of passive pedestrian protection and occupant protection, respectively, whereas the structural strains of 0.05–0.2 and 0.23–0.5 are set as the corresponding energy absorption ranges (Figure 5c). Based on the objective function, a dual‐platform energy‐absorbing structure is obtained (Figures S14 and S15, Supporting Information). When a vehicle collides with a pedestrian, the pedestrian receives a small reaction force and the structure exhibits a stable energy absorption effect because only the peripheral rods are deformed (Figure 5cI). When a collision occurs between cars, the rapid contact between the inner and outer rods of the structure increases the stress of platform and ensures sufficient energy absorption effect (Figure 5cII,III). The lattice structure was verified experimentally (Figure 5c,d) with an experimental error of 15.88%. The optimization process and deformation process of the structure is shown in Figures S14 and S15, Supporting Information. For practical applications, the lattice structure can be inserted into the bumper to improve the energy absorption protection effect of the car.^[^ ^1b,c^ ^]^

In this paper, a 2D periodic structure is selected as the design object, which can be easily extended to 3D by updating the algorithm. Zhang et al.^[^ ^17^, ^30^ ^]^ show that the gradient lattice collapses layer by layer under compression. The research of Liu and Li shows that the spatial inhomogeneous structure also has the ability to improve energy absorption efficiency.^[^ ^31^ ^]^ Inspired by these studies, the design objects of our method are not limited to periodic structures, and more structural designs based on the stress‐strain curve are expected to be obtained by multiscale or gradient optimizations. The proposed method also has broad applicational prospects in dealing with material nonlinearity and geometric nonlinearity, and more meaningful work is worth exploring. The application of structures in this paper is aimed at medium and low‐speed impact, so quasi‐static loading is selected to carry out compression experiments. The method presented in this paper can also be applied to the design of energy absorbing structures under high‐speed impact, by considering the strain rate effect of parent material.

## 3 Conclusions

We have proposed an inverse design method for energy‐absorbing structures of mechanical metamaterials based on genetic algorithms. This method not only realizes the design of structures with ideal energy‐absorbing curves but also realizes the design of structures with specific‐shaped energy‐absorbing curves. The optimized structural properties are verified by compression experiments. We have also demonstrated the feasibility and practicability of the algorithm for functional energy‐absorbing structures by taking the energy‐absorbing protection of automobiles. Most importantly, the design of various energy‐absorbing curve structures can be realized through the search for structure, which greatly reduces the difficulty of structural design. Our optimization framework provides a new solution path for the elastic‐plastic large deformation problem that was unable to be resolved by using conventional gradient algorithms or genetic algorithms, and simplifies the design process of energy‐absorbing mechanical metamaterials.

## 4 Experimental Section

### Optimization Settings and Model Evaluation

Herein, the model design domain size was 100 × 100 and the finite element model size was 100 mm×100 mm. The population size was set to be 50 and the termination condition was set to be structural stress exceeding 1.5 times the target *σ_m_*. The contour coordinate points of the structure were extracted, and the mutation operation was realized by changing the material distribution near each coordinate point. Two mutation modes were defined: contour extension and outline contraction. The contour expansion was realized by adding materials outward from contour points, while the contour contraction was realized by reducing materials inward from contour points. The probabilities of these two mutation modes at each coordinate point were *B*_rate1_ and *B*_rate2_ (Figure S4f, Supporting Information) respectively. The probability *B*_rate__1_ (Figure S4f, Supporting Information) of structure outline expansion was set to be 0.05, and the probability *B*_rate__2_ (Figure S4f, Supporting Information) of structure outline contraction was set to be 0.01. The crossover probability was set to 1.0, and each case was optimized for 100 generations. In the case of *σ_m_* = 10 MPa, the minimum thickness was set to be 3% of the side length, and the minimum thickness of the other case was set to be 6% of the side length. *O* *b* *j*_1_ was set as *ε_max_* – *ε* to optimize the densification strain of the structure. The smaller value of *Obj*_1_ resulted in a better compressive performance of the structure. *Obj*_2_ was set to be $\left(\int \right|\sigma^{*}-\sigma^{\mathrm{obj}}\left|d\varepsilon \right)/\left(\int \sigma^{\mathrm{obj}}d\varepsilon \right)$ and the difference between the structure and target stress‐strain curve was integrated and normalized. The nondominated sorting algorithm only distinguished the model by rank and the crowding degree could not judge the optimal structure in the same rank. To facilitate the presentation of results, Equation (1) was defined to evaluate the Score of the objective function of each model:

(1) $${\mathrm{Score}}_{\mathrm{ij}}=1\times \frac{{\mathrm{Obj}}_{1}^{\mathrm{ij}}}{0.8}+3\times {\mathrm{Obj}}_{2}^{\mathrm{ij}}$$

where ${\mathrm{Obj}}_{1}^{\mathrm{ij}}$ is the difference between the designed densification strain and the target strain 0.8, and the first term of the equation is divided by 0.8 for normalization. The ${\mathrm{Obj}}_{2}^{\mathrm{ij}}$ is the difference between the designed stress‐strain curve and the target stress‐strain curve divided by the target strain energy. Coefficient 1 of the first term and the coefficient 3 of the second term were used to give the relative weights of the two objective functions in the total scores. The index *i* represents the generation number and inde *j* refers to the serial number of individuals in each generation. The individual with the smallest score value in each generation corresponds to the optimal structure.

### Finite Element Analysis Modeling

The mesh size convergence is shown in Figure S7a, Supporting Information, and the stress‐strain curves were almost consistent when the mesh size was less than or equal to 1.0 mm. Therefore, the mesh size of all models was set to 1.0 mm. Periodic boundary conditions were applied to the model, and four rigid plates were applied to implement coupled kinematic constraints. The existence of rigid plate makes the internal structure having symmetry constraints instead of being free, which is consistent with the deformation during the compression process. As shown in Figure 2aVII, the left and lower plates (solid green lines) have fixed constraints, where purple triangles are translational constraints and red dots are rotational constraints. The upper plate was coupled to the motion of Master Node 2, and the degrees of freedom in the *x*‐direction were constrained. The right plate was coupled to the motion of Master Node 4 and the degrees of freedom in the *y*‐direction are constrained. There was no contact interaction between different plates. The hard contact was chosen for interaction between the plate and the structure, as well as the self‐interaction within the structure. A vertical displacement was set along the *y*‐direction to simulate the compression process, as shown by the black‐colored arrows in Figure 2aVII. The design target of the structure is the stress‐strain curve when the structure is compressed in the *y*‐direction, and the unit cell only has 1/4 symmetry and periodic constraints. Therefore, the objective function and constraints do not require the unit cell to be elastic isotropic.

Buckling analysis was carried out to obtain the initial imperfection factors, in which the first ten order eigenmode buckling modes were selected as representative buckling modes and introduced into the finite element model. The buckling eigenvalue of all structure are far greater than the initial peak force, and the compression simulation results of model with imperfection and without imperfection are consistent. Therefore, the main reason for the initial failure of the structure is plastic yield rather than elastic buckling. The detailed analysis is attached to Section S2 of Supporting Information.

A 64‐core workstation, with CPU INTEL Xeon Platinum 8358, was selected for calculations. The optimal number of cores for jobs was 8 and the number of jobs running at the same time was 8. The calculation time of each generation was 30 to 60 min, and the optimal structure can be obtained in 1 to 3 days.

### Experimental Section

The material was 304 stainless steel, whose true stress‐strain curve is shown in Figure S7b, Supporting Information. A lattice of 5 × 3 cells was chosen to represent the periodic structure and 304 stainless steel powder was used to print the test specimen using L‐PBF, as shown in Figure 4a‐d and Figure 5cV. The detailed printing parameters are shown in Table S1, Supporting Information. The test specimen size was 80 mm × 48 mm × 10.9 mm, and the minimum feature dimension of each specimen (*σ_m_* = 10, 20, 40, 60 MPa and double platform) was 1.09 mm, 1.2 mm, 1.28 mm, 1.51 mm, and 1.54 mm, respectively. The DIC experiments were carried out using a universal testing machine and video extensometer. The upper and lower indenters were marked with line probes for accurate displacement measurements and the deformation process of the structure was recorded using a high precision camera in the video extensometer.

## Conflict of Interest

The authors declare no conflict of interest.

## Supporting information

Supporting Information

## Data Availability

The data that support the findings of this study are available from the corresponding author upon reasonable request.

## References

[1] a) J. Rivera , M. S. Hosseini , D. Restrepo , S. Murata , D. Vasile , D. Y. Parkinson , H. S. Barnard , A. Arakaki , P. Zavattieri , D. Kisailus , Nature 2020, 586, 543; 33087910 10.1038/s41586-020-2813-8

[2] a) Z. Fan , G. Lu , K. Liu , E. Struct. 2013, 55, 80;

[3] F. C. Bardi , S. Kyriakides , Int. J. Mech. Sci. 2006, 48, 830.

[4] H. Yang , H. Lei , G. Lu , Thin‐Wall Struct. 2021, 160, 107380.

[5] G. H. Daneshi , S. J. Hosseinipour , Struct. Mater. 2002, 11, 289.

[6] E. T. Filipov , T. Tachi , G. H. Paulino , Proc. Natl. Acad. Sci. USA 2015, 112, 12321. 26351693 10.1073/pnas.1509465112 PMC4603468

[7] J. Ma , H. Dai , S. Chai , Y. Chen , Mater. Des. 2021, 206, 109808.

[8] K. Liu , R. Sun , C. Daraio , Science 2022, 377, 975. 36007025 10.1126/science.abn1459

[9] Z. Jia , Y. Yu , S. Hou , L. Wang , J. Mech. Phys. Solids 2019, 125, 178.

[10] F. Barthelat , H. Tang , P. Zavattieri , C. Li , H. Espinosa , J. Mech. Phys. Solids 2007, 55, 306.

[11] N. S. Ha , G. Lu , Composites, Part B 2020, 181, 107496

[12] J. Li , Z. Chen , Q. Li , L. Jin , Z. Zhao , Adv. Sci. 2022, 9, 2105769. 10.1002/advs.202105769 PMC9069190 35257516

[13] a) M. Y. Wang , S. W. Zhou , Comput. Model. Eng. Sci. 2004, 6, 469;

[14] a) H.‐W. Dong , S.‐D. Zhao , X.‐B. Miao , C. Shen , X. Zhang , Z. Zhao , C. Zhang , Y.‐S. Wang , L. Cheng , J. Mech. Phys. Solids 2021, 152, 104407;

[15] a) J. Wu , A. Clausen , O. Sigmund , Comput. Methods Appl. Mech. Eng. 2017, 326, 358;

[16] a) J. Wu , Z. Luo , H. Li , N. Zhang , Comput. Methods Appl. Mech. Eng. 2017, 319, 414;

[17] a) J. P. Groen , F. C. Stutz , N. Aage , J. A. Bærentzen , O. Sigmund , Comput. Methods Appl. Mech. Eng. 2020, 364, 112979,;

[18] F. Wang , O. Sigmund , J. S. Jensen , J. Mech. Phys. Solids 2014, 69, 156.

[19] A. Clausen , F. Wang , J. S. Jensen , O. Sigmund , J. A. Lewis , Adv. Mater. 2015, 27, 5523. 26291030 10.1002/adma.201502485

[20] a) P. Kumar , C. Schmidleithner , N. B. Larsen , O. Sigmund , Struct. Multidiscip. Optim. 2020, 63, 1351;

[21] a) J. Kato , H. Hoshiba , S. Takase , K. Terada , T. Kyoya , Struct. Multidiscip. Optim. 2015, 52, 507;

[22] F. Wang , B. S. Lazarov , O. Sigmund , J. S. Jensen , Comput. Methods Appl. Mech. Eng. 2014, 276, 453.

[23] S. Y. Wang , K. Tai , Comput. Methods Appl. Mech. Eng. 2005, 194, 3749.

[24] B. Xu , J. S. Jiang , J. P. Ou , J. Sound Vib. 2007, 307, 393.

[25] a) B. S. Mekki , J. Langer , S. Lynch , Int. J. Heat Mass Transfer 2021, 170, 121002;

[26] B. Xu , J. P. Ou , J. S. Jiang , Finite Elem. Anal. Des. 2013, 64, 1.

[27] S. Y. Wang , K. Tai , M. Y. Wang , Int. J. Numer. Methods Eng. 2006, 65, 18.

[28] K. Deb , A. Pratap , S. Agarwal , T. Meyarivan , IEEE Trans. Evol. Comput. 2002, 6, 182.

[29] Q. Zeng , Z. Zhao , H. Lei , P. Wang , Int. J. Mech. Sci. 2022, 240, 107920.

[30] H. Zhang , Y. Wang , Z. Kang , Int. J. Eng. Sci. 2019, 138, 26.

[31] a) P. Liu , Z. Kang , Y. Luo , Addit. Manuf. 2020, 36, 101427;

