# Supplementary material for: Inverse Design of Energy‐Absorbing Metamaterials by Topology Optimization
Source: Adv Sci (Weinh). 2022 Dec 11;10(4):2204977. doi: 10.1002/advs.202204977 (PMC9896075; doi:10.1002/advs.202204977)
Supplement: Supplementary file 1 — Supporting Information [file ADVS-10-2204977-s001.pdf]

Supporting Information

Inverse Design of Energy-Absorbing Metamaterials by Topology Optimization

Qingliang Zeng, Shengyu Duan, Zeang Zhao*, Panding Wang, Hongshuai Lei**

This PDF file includes:

- Supporting text information.
- Figure S1. NSGAII algorithm implementation process.
- Figure S2. Improved algorithm flow.
- Figure S3. Structural symmetry and gene expression.
- Figure S4. Problems with traditional genetic strategies.
- Figure S5. Structural thickness control and geometric feature extraction.
- Figure S6. Parallel computing model.
- Figure S7. Materials and model parameters.
- Figure S8. Target stress-strain curve.
- Figure S9. The optimization process of the optimal structure with the same platform stress.
- Figure S10. Deformation process of optimal structure with the same platform stress under compressive loads.
- Figure S11. The deformation process of an optimal structure under a compressive load.
- Figure S12. The optimization process of the high platform stress.
- Figure S13. The deformation process of the optimal structure under a compressive load.
- Figure S14. The optimization process of optimal structure with double platform stress.
- Figure S15. Deformation process of double platform energy absorption structure.
- Figure S16. Buckling analysis of unit cells.
- Table S1. The printing parameters of the test specimen.
- Table S2. Design errors of structure with the same platform stress.
- Table S3. Simulation and experimental errors of an ideal energy-absorbing structure.

S1 Materials and Methods

S1.1 Stiffness Matrix Tunable Topological Optimization Algorithm

The optimization efficiency and results of the genetic algorithm are strongly dependent on the initial population. Hence, structures with different moduli and densities are required to guide the algorithm optimization. A stiffness-matrix tunable topology optimization algorithm was proposed in our previous work, [29] where the design objective was set as the structural equivalent stiffness matrix and the constraint function was the material volume fraction.

Based on the homogenization theory, the structural equivalent stiffness matrix can be expressed as:

$$E_{ijkl}^{*} = \frac{1}{|\varphi_m|}\int_{\Omega_m} E_{pqrs}\left(\varepsilon_{pq}^{0(ij)} - \varepsilon_{pq}^{(ij)}\right)\left(\varepsilon_{rs}^{0(kl)} - \varepsilon_{rs}^{(kl)}\right)d\varphi_m \tag{S1}$$

where E_{ijkl}^{*} refers to the equivalent stiffness matrix and φ_m represents the volume or area of the microstructure. $\varepsilon_{pq}^{0(ij)}$ denotes the unit test strain field and ε_{pq}^{ij} refers to the structural response corresponding to the test strain field.

After homogenization equivalence, the objective function of topological optimization can be expressed by structural properties. The concept is based on the SIMP method combined with a matrix of weight coefficients to build the objective function. The optimization objective is to achieve an equivalent stiffness matrix consistent with the target stiffness matrix. The constraints are made of the balance equation under periodic conditions, volume constraints and design variable constraints. The specific expression of the optimization problem can be expressed according to Equation (S2):

$$\begin{cases} Find: \rho = (\rho_1, \ldots \rho_N) \\ Min: C = \frac{1}{2}\sum_{i,j,k,l=1}^{d} w_{ijkl}\left(Q^{*}_{ijkl} - Q^{H}_{ijkl}\right)^2 \qquad d = 2 \\ S.t: \begin{cases} KU^{A(kl)} = F^{(kl)} \qquad k,l = 1,\ldots\ldots,d \\ \sum_{e=1}^{N} v_e \rho_e / |Y| \le v \\ 0 \le \rho_e \le 1 \qquad\qquad e = 1,\ldots N \end{cases} \end{cases} \tag{S2}$$

where ρ denotes the design variable and w_{ijkl} represents the weighting factor. Q^{*}_{ijkl} and Q^{H}_{ijkl} refer to the structural equivalent and target stiffness matrices, respectively. v_e refers to the volume of grid element and v represents the volume fraction of design material.

The range of the relative Young's modulus and the relative shear modulus is calculated to satisfy the Hashin-Shtrikman bounds according to the relative density of the structure during the initial design optimization. Therefore, the target stiffness matrix satisfies the Hashin-Shtrikman bounds.

Fifty structures with relative Young's modulus in the range of 0 to 0.2 and relative density in the range of 0 to 0.8 are obtained based on this algorithm, which is selected as the initial population, as shown in Figure 2aI.

S1.2 NSGAII Algorithm

The energy-absorbing structure is designed for multi-objective optimization. Hence, NSGAII in the genetic algorithm is selected as the basic algorithm. As shown in Figure S1a, it is the main flow of genetic algorithm. Elite individuals are the parent of the population and children are generated by crossover and mutation operations, as shown in Figure S1c and S1d. The children are subjected to fitness evaluation, as shown in Figure S1b, forming a non-dominated ranking. The elite individuals of the sorted children are retained as the parents of the next generation.

S1.3 Genetic Algorithms for Energy-Absorbing Topology Optimization

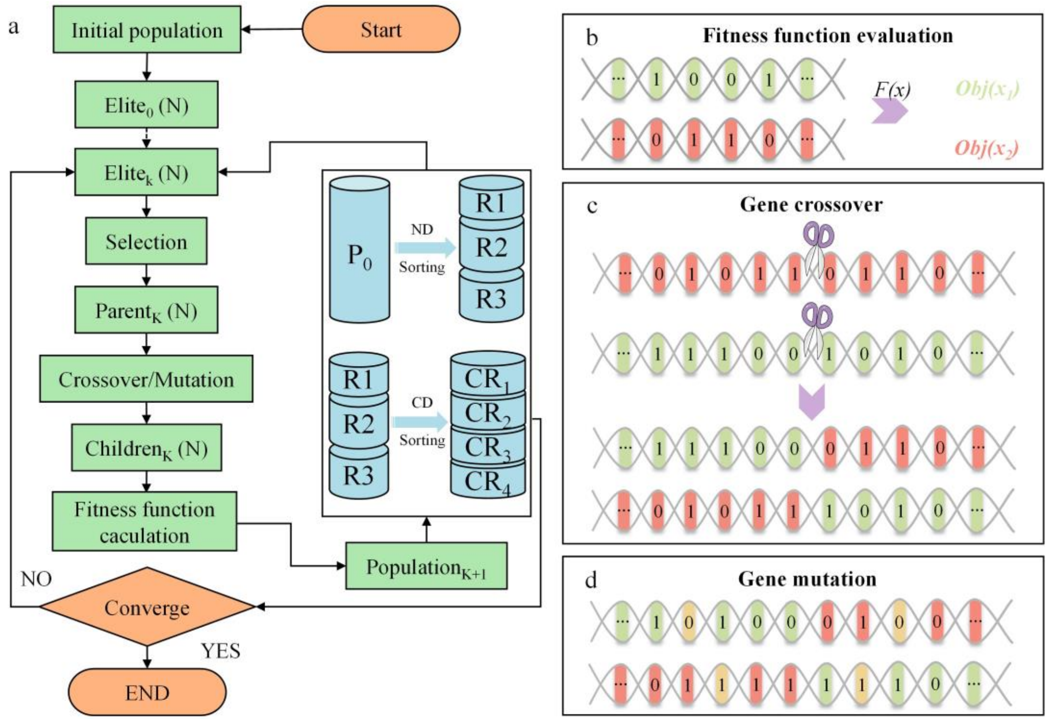

Figure S1. NSGAII algorithm implementation process. a) Algorithm flow. b) Fitness function evaluation, where different colors represent individuals with different genes and *F(x)* represents the finite element analysis; c) Gene crossover, where the cut place is the selected intersection and the gene is exchanged to form a new individual; and d) Gene mutation, which occurs in individuals after crossover and mutated genes are shown in yellow color.

A genetic strategy suitable for the optimization of porous materials is proposed based on the NSGAII algorithm. Unlike the sorting process in the original algorithm, the parent and children are sorted together in the new algorithm (Figure S2). Hence, the best genes are preserved as much as possible. The structures are processed symmetrically (Figure S3a and S3b) and finally formed the gene individual representation, as shown in Figure S3c. When performing the intersection of porous structures, the intersection selection scheme of the original algorithm is completely random and does not perform secondary evaluation, leading to three types of problems in the crossed structure (Figure S4a, S4b and S4c).

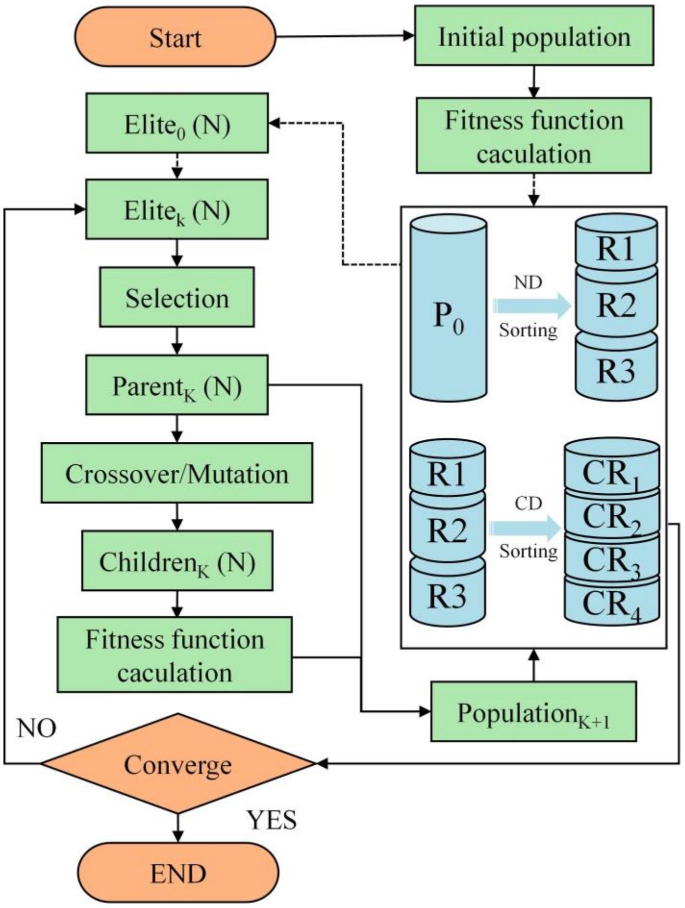

Figure S2. Improved algorithm flow.

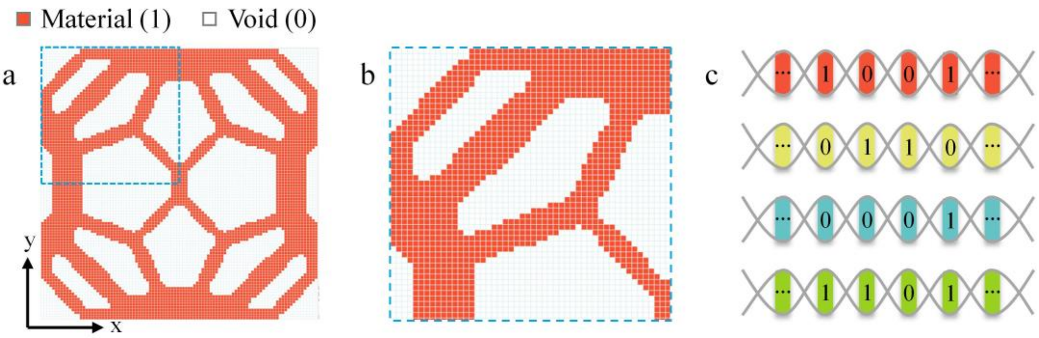

Figure S3. Structural symmetry and gene expression. a) Design domain with a grid of pixels, where the structures are shown in red and holes are shown in white; b) Symmetric processing; c) Gene representation, where individual structures are converted to row vectors as genes and different colors represent individual genes.

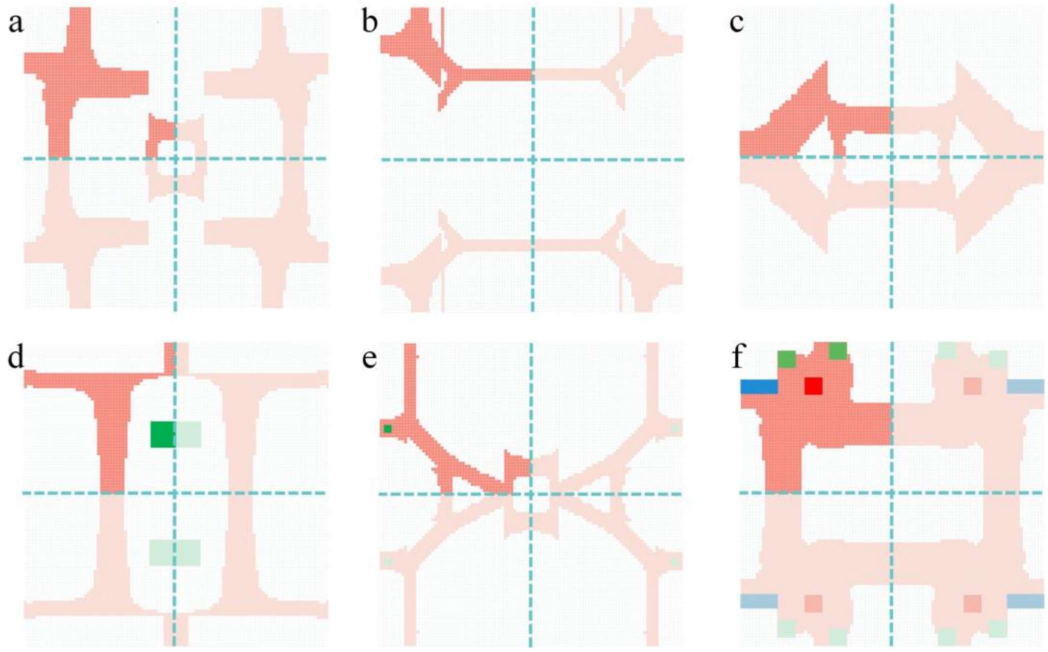

Figure S4. Problems with traditional genetic strategies. a) Cross discontinuity, where intersected structures cannot form a complete structure and the intersection is the structural breakpoint. The blue dashed line represents the symmetry axis and the dashed part represents the symmetrical structure. b) Symmetric discontinuity, where the crossed structure is continuous and it cannot form a complete structure after symmetry. c) Periodic discontinuity, where intersected and symmetrical structures are continuous, and periodic boundary conditions cannot be imposed on such structures; d) Deleterious mutation, where random mutations make isolated structures appear in the design domain and the original structure cannot be calculated because the continuity is broken. e) Null mutation, where small defects inside the structure bring grid disaster to the calculations. f) The proposed outline mutation, where the increase in material at the outline is indicated in blue, the decrease in material at the outline is indicated in green and the appearance of large-sized voids inside the structure is indicated in red.

The discontinuous structure cannot be evaluated for fitness and exhibits limited effect on population genetic improvement. In the improved algorithm, the continuity and symmetry are calculated after the configuration crossover. The cross point is re-selected if the structure is not continuous and, if the continuous structure cannot be obtained after re-selection 50 times, the parent is re-selected to ensure that the crossover structure is effective. The random single-point mutation method adopted by the traditional algorithm makes the structure appear harmful and invalid mutations, as

shown in Figure S4d, and S4e, resulting in a discontinuous or unanalyzable structure. Therefore, an outline variation scheme for porous structures is proposed, as shown in Figure S4f. The boundary points of the structure are picked by the outline recognition algorithm, and two mutation operators, i.e., B_{rate1} and B_{rate2}, are defined. The mutation module expands the outline of structure according to the value of B_{rate1} and contraction the outline of the structure according to the value of B_{rate2}. The validity of structure is guaranteed by precise variation of the profile of porous structure.

In addition, thickness constraints are imposed to ensure that the structure is easy to manufacture. The centerline of the structure is extracted by the skeleton algorithm, as shown in Figure S5a-c, and the minimum thickness of the structure is controlled by setting the size of skeleton pixel expansion. The final configuration is formed by the superposition of the expanded structure and original structure. Moreover, the minimum thickness of the structure is lifted and the characteristics of other regions are preserved. This is the genetic improvement scheme proposed for the porous structure based on the original algorithm.

The grid of design domain is a pixel grid, and small features in the local area causing non-convergence of the finite element analysis. This problem is effectively solved if the design grid is decoupled from the computational grid. As shown in Figure S5d-f, the outline recognition algorithm is used to obtain structural outline and geometric model is obtained by interpolation. The structural mesh is generated on the geometric model, and the size and angle of mesh can be adjusted to improve mesh quality. The features in the design domain are simplified to the features in the geometric model. Based on the mesh decoupling method, the model accuracy is preserved and computational difficulty is reduced. This solves the difficulty of forming a finite element model from the design model.

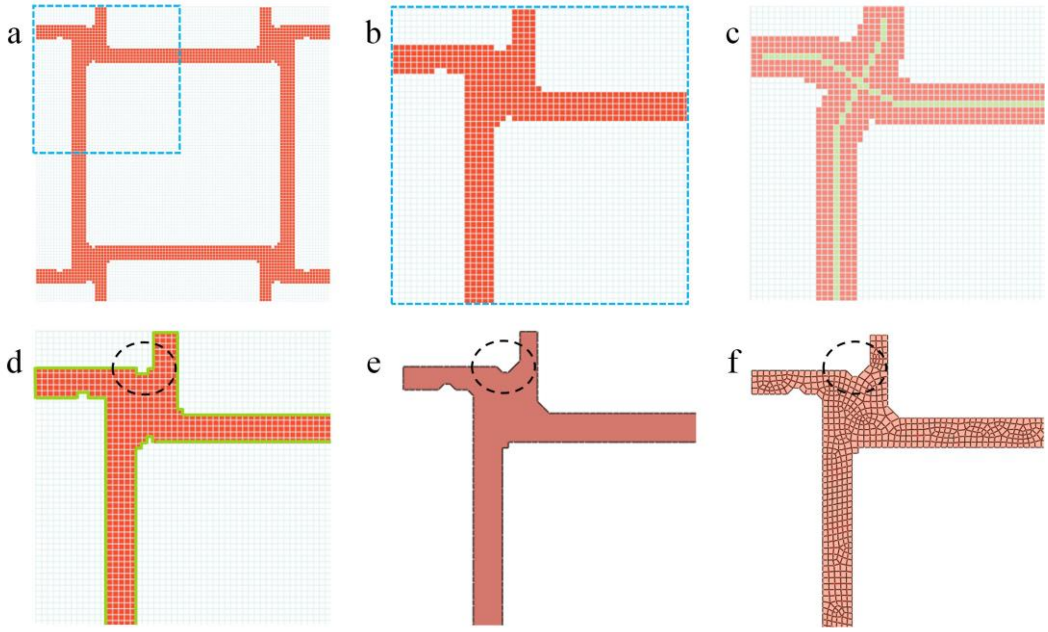

Figure S5. Structural thickness control and geometric feature extraction. a) Designed structure. b) Simplified structure. c) Size control, where the centerline is represented by the green line and the structure is final configuration after superposition. d) Structural outline recognition, where recognized outlines are indicated by green lines and features of small size are marked by black dashed lines. e) Geometric model, where the fitted coordinates are formed using line interpolation based on the outline coordinates and generate the geometric model. f) Mesh model, which is generated based on the geometric model and features of the geometric model are completely preserved.

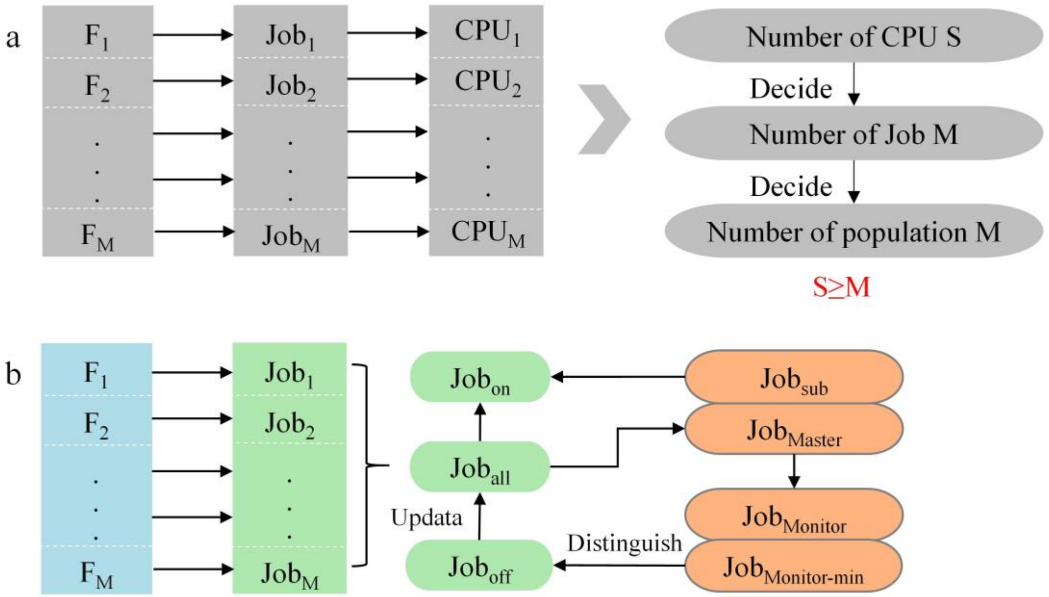

Figure S6. Parallel computing model. a) simultaneous submission model. b) queuing model.

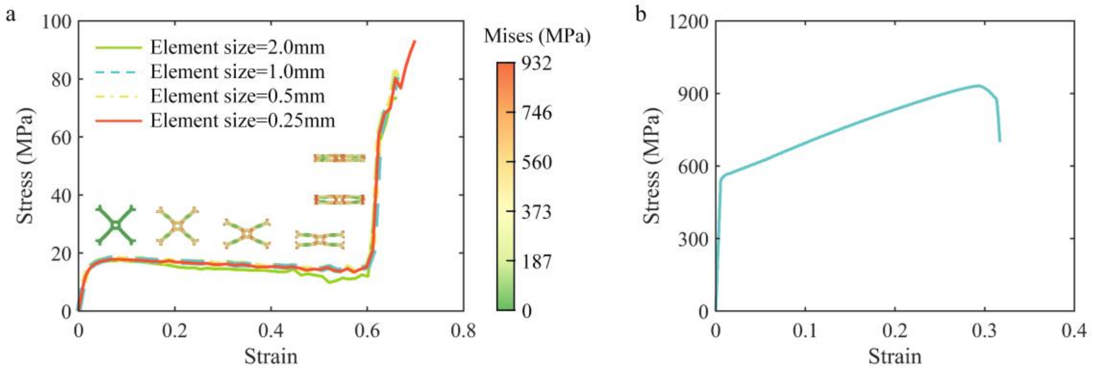

Figure S7. Materials and model parameters. a) Stress-strain curves under different mesh sizes. b) True stress-strain curve of the 304 stainless steel.

Each individual in the population is an independent computing job, and a reasonable and balanced allocation of computing resources is the key to efficient computing. In general, the parallel strategy of genetic algorithm analysis is divided into following two types: sequential submission and simultaneous submission. In a sequential commit strategy, computing resources are focused on completing a single job until completion. Frustratingly, for jobs with a small amount of computation, computing efficiency is not strictly positively related to computing resources. Too many CPU cores increase computation scheduling time and reduce efficiency, which is the reason of the software existed upper limit of accelerated cores number. The simultaneous submission strategy (Figure S6a) assigns each job to a single CPU for execution, which leads to the fact that the number of physical cores determines the number of simultaneously submitted jobs and, thus, the population. Design variables being limited by hardware is a catastrophic problem for structural design, and a single core is not necessarily the optimal number of cores for the computing job. The calculation time of each generation depends on the time to complete the most complex job, and the difference in the calculation time of each task inevitably leads to a large number of CPUs being idle.

A queuing model is proposed to solve the problems of existing parallel schemes, as shown in Figure S6b. Before parallel computing, the optimal number of cores for this type of job calculation is determined by computing multiple cases with the number of cores and model as variables. When parallel computing starts, the program Job_{master}

divides all tasks into Job_{all} uniformly and calls the program Job_{sub} to submit tasks according to the preset optimal number of cores and number of simultaneous computing jobs. The successfully submitted jobs are assigned the Job_{on} attribute and $Job_{monitor}$ determines whether the Job_{on} has completed the calculations. The program $Job_{monitor-min}$ has a function of extracting real-time calculation results and jobs that meet the conditions are terminated earlier rather than complete analysis. Jobs that fully compute or terminate computation are screened out by Job_{master} in real time and updated to Job_{off}. Job_{master} updates the Job_{all} according to the specific number of Job_{off} and submits a new Job_{on}. The task cycle monitoring and submission continue until the analysis is completed. The queuing model achieves nearly 100% CPU utilization and genetic algorithm optimization of any number of populations can run on computers with any core configuration, removing the hardware limitation of the algorithm. This model improves computational efficiency.

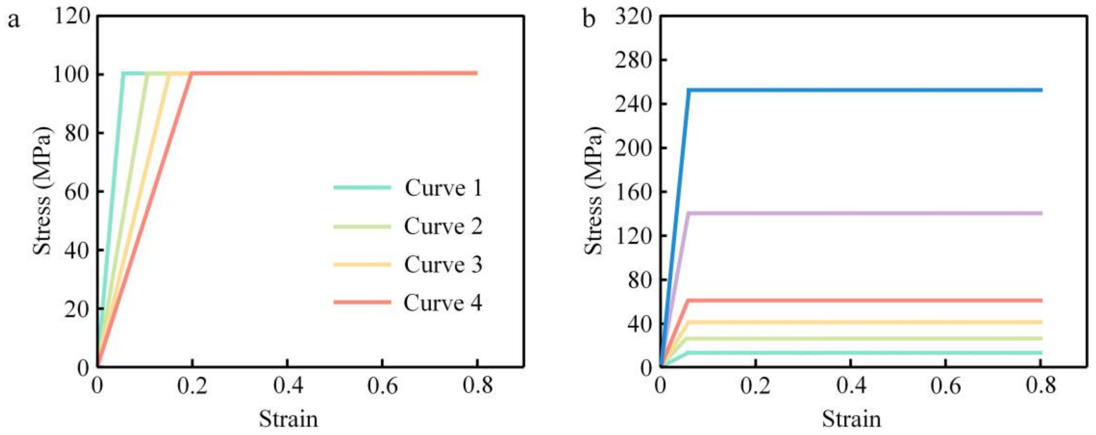

Figure S8. Target stress-strain curve. a) Different energy absorption curves with the same platform stress. b) Energy absorption curves with different platform stresses.

S2 SUPPLEMENTARY RESULTS

The design of energy absorbing structures for different target curves (curve1, curve2, curve3 and curve4) for the same platform stress was carried out to verify the diversity of the algorithm, as shown in Figure S8a. The optimization process of the optimal structure is shown in Figure S9 and the deformation process of the structure is shown in Figure S10. The design error of the structure is shown in Table S2. The given examples provide the design of ideal energy-absorbing structures and the design

of functional energy-absorbing structures. The ideal energy absorption design is an energy absorption structure with different plateau stresses, i.e., σ_m = 10, 20, 40, 60, 140 and 250 MPa, as shown in Figure S8b. The optimal structural optimization process for low plateau stress (σ_m = 10, 20, 40 and 60 MPa) is shown in Figure 3 and the deformation process of the structure is shown in Figure S11. The optimization process of the optimal structure with high platform stress (σ_m = 140 and 250 MPa) is shown in Figure S12, and the deformation process of the structure is shown in Figure S13. The simulation error of the unit cell, and simulation and experimental errors of the lattice are compared with the design target, as shown in Table S3. In the design of functional energy-absorbing structure, the optimization process for optimal structures with double platform stress is shown in Figure S14 and the deformation process of the structure is shown in Figure S15. The simulation error of the unit cell is found to be 8.38%, the simulation error of the lattice is found to be 15.34%, and the experimental error of the lattice is found to be 15.88%.

Buckling analysis was carried out to investigate the initial failure mode of the structure. The first mode of the structure is shown in Figure S16a, and the buckling eigenvalue is far greater than the initial peak force. Therefore, the structure was collapse directly rather than buckle during compression. The compression simulation results of model with imperfection and without imperfection are shown in Figure S16b. The consistent curves of the two models also indicate that the introduction of initial imperfection factors has no effect on the structural failure process. Other structures in the manuscript were also buckling analyzed, and the same conclusion was obtained.

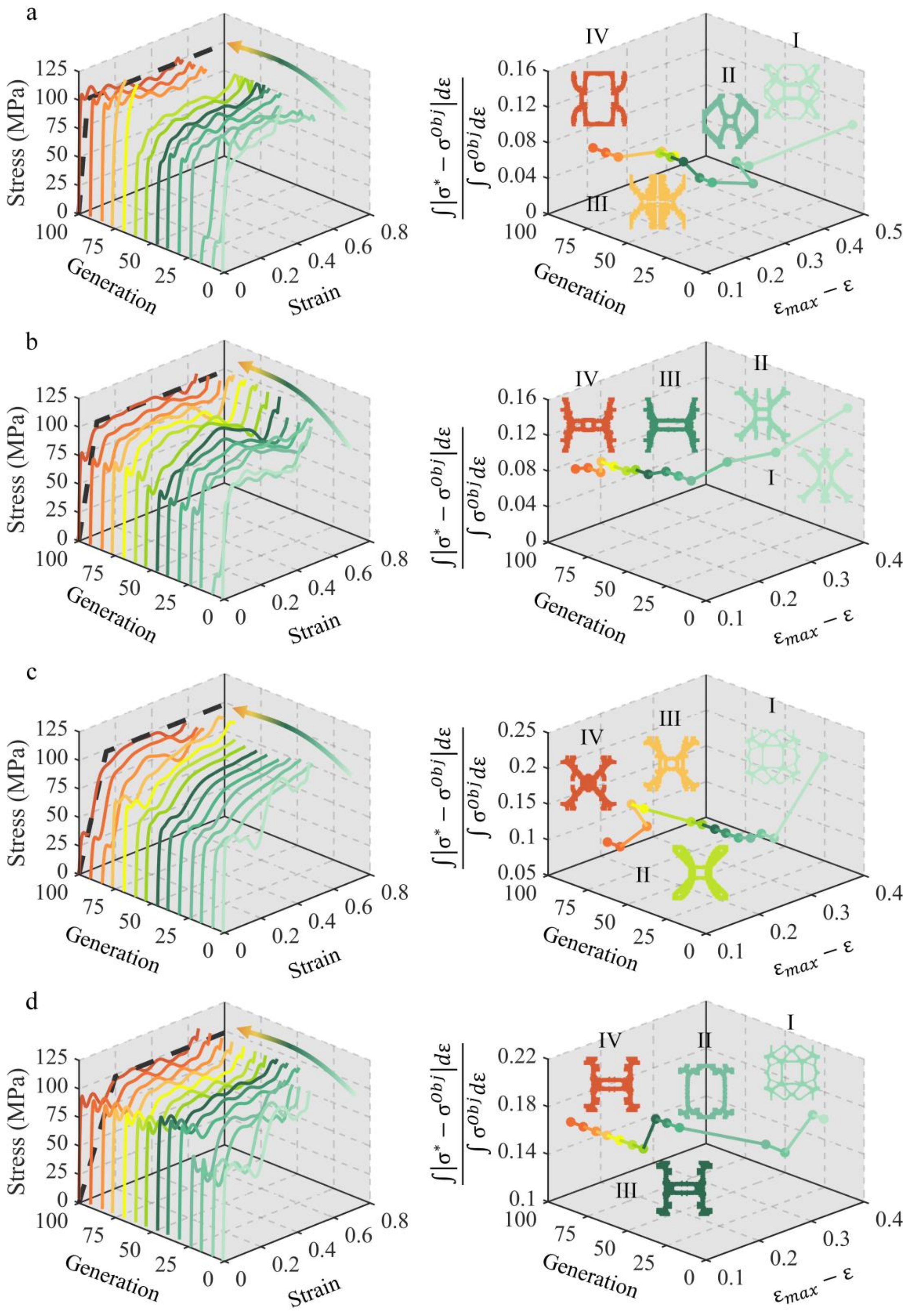

Figure S9. The optimization process of the optimal structure with the same platform stress. a) ε_s = 0.05. b) ε_s = 0.1. c) ε_s = 0.15. d) ε_s = 0.2.

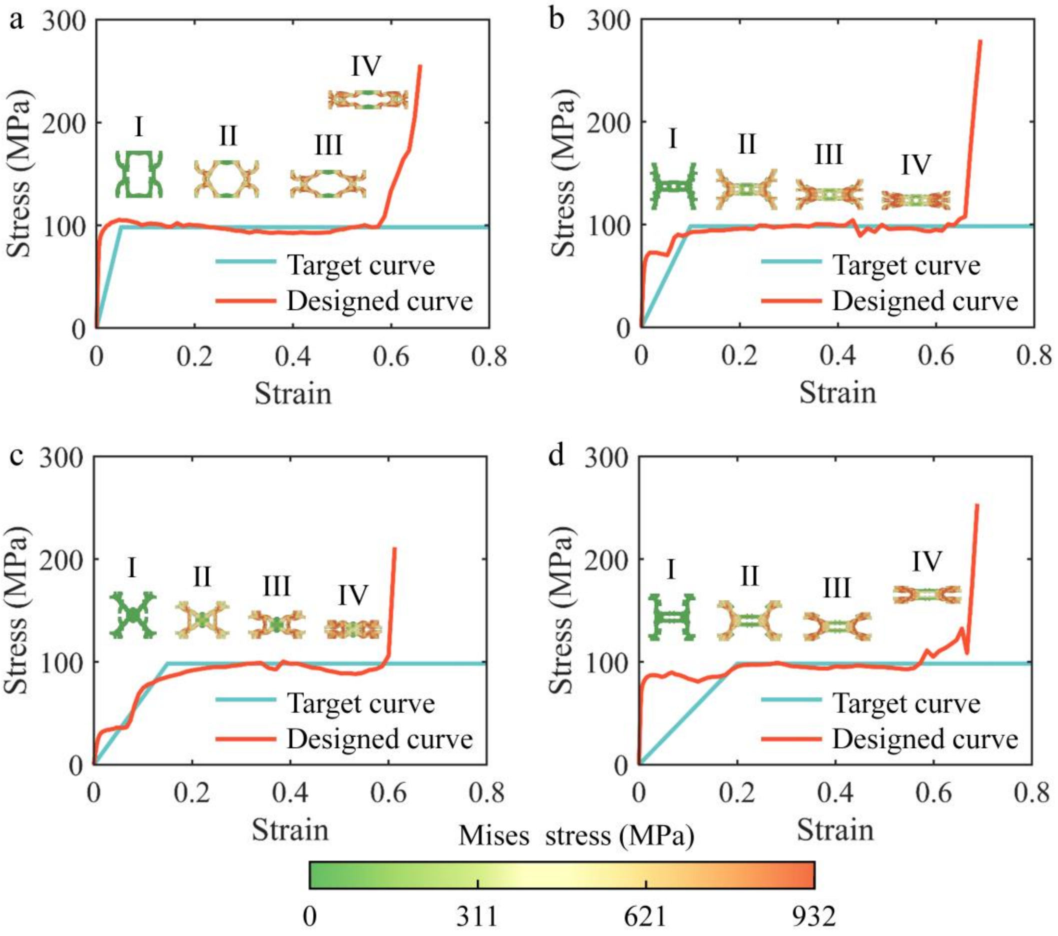

Figure S10. Deformation process of optimal structure with the same platform stress under different compressive loads. a) $\varepsilon_s = 0.05$. b) $\varepsilon_s = 0.1$. c) $\varepsilon_s = 0.15$. d) $\varepsilon_s = 0.2$.

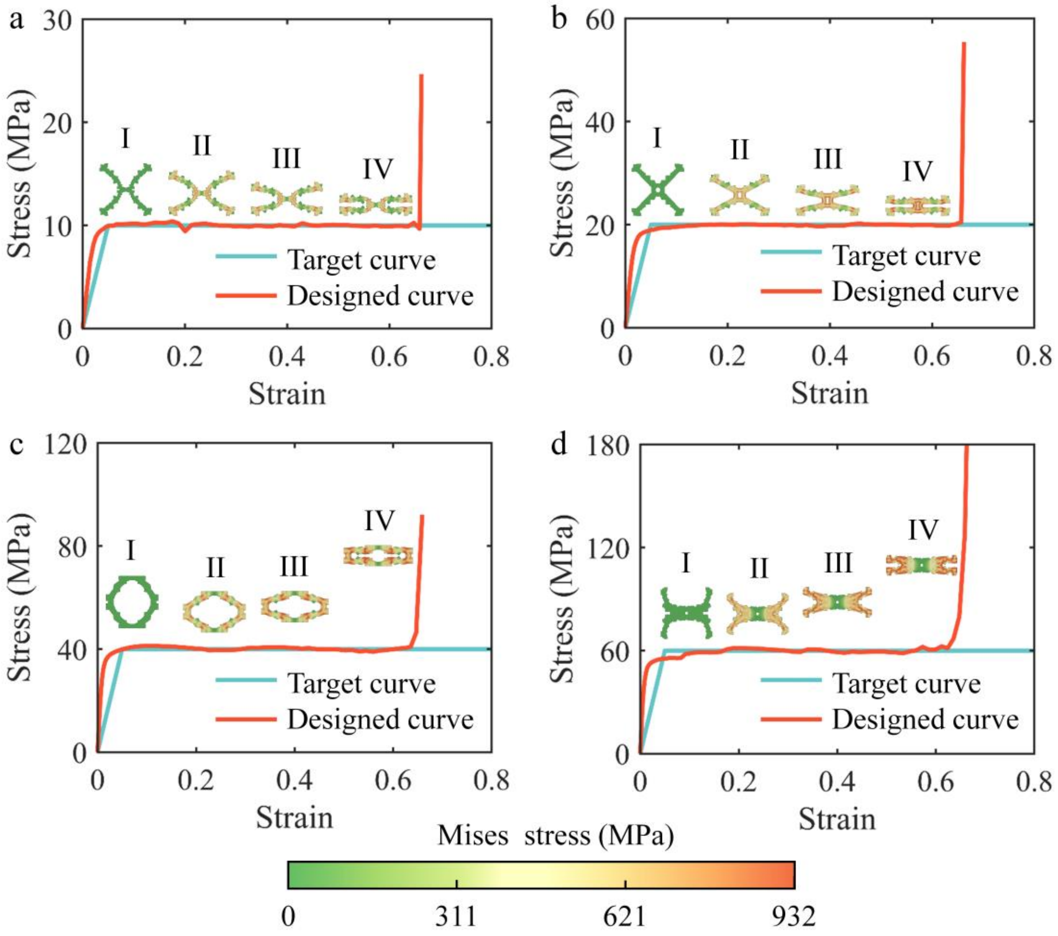

Figure S11. The deformation process of an optimal structure under a compressive load of a) σ_m = 10 MPa, b) σ_m = 20 MPa, c) σ_m = 40 MPa, and d) σ_m = 60 MPa, where I, II, III and IV represent the deformation state of the structure at the strain of 0, 0.2, 0.4 and 0.6, respectively.

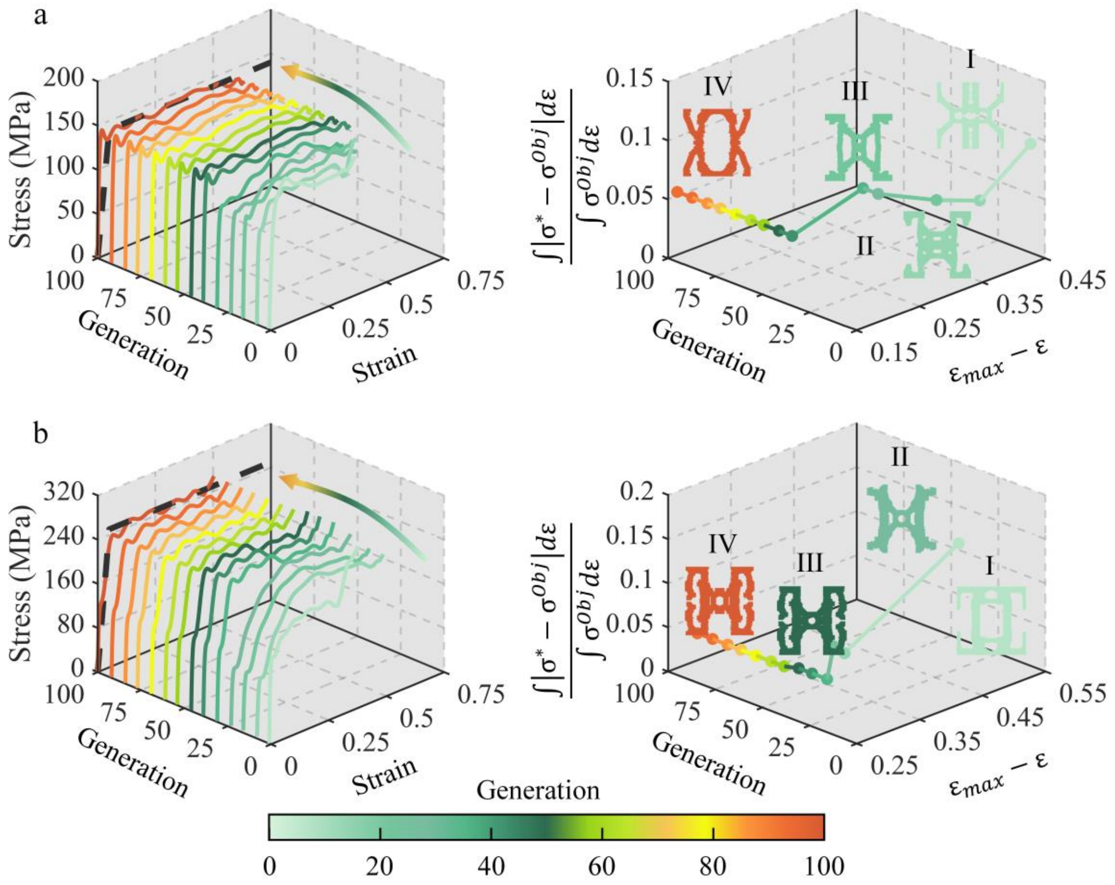

Figure S12. The optimization process of the high platform stress. a) σ_m = 140 MPa and b) σ_m = 250 MPa.

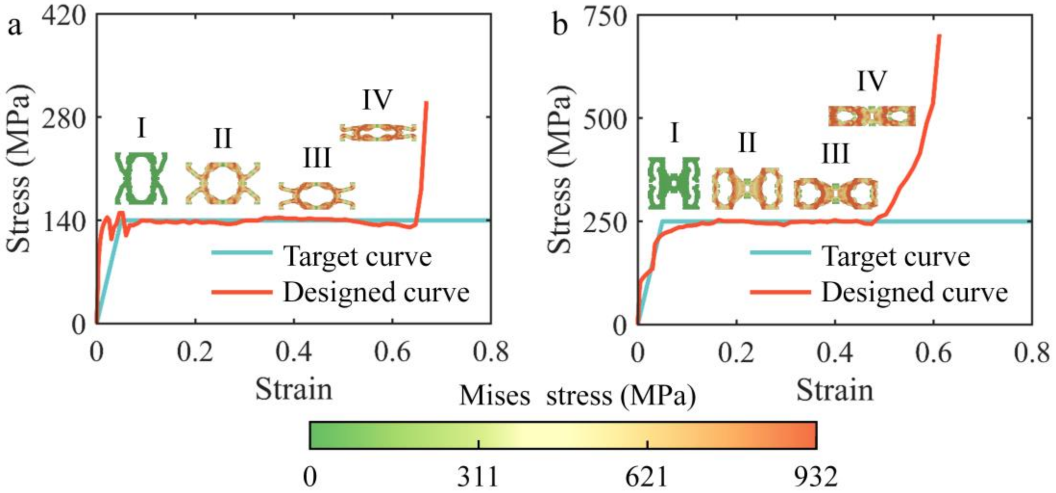

Figure S13. The deformation process of the optimal structure under a compressive load of a) σ_m = 140 MPa and b) σ_m = 250 MPa.

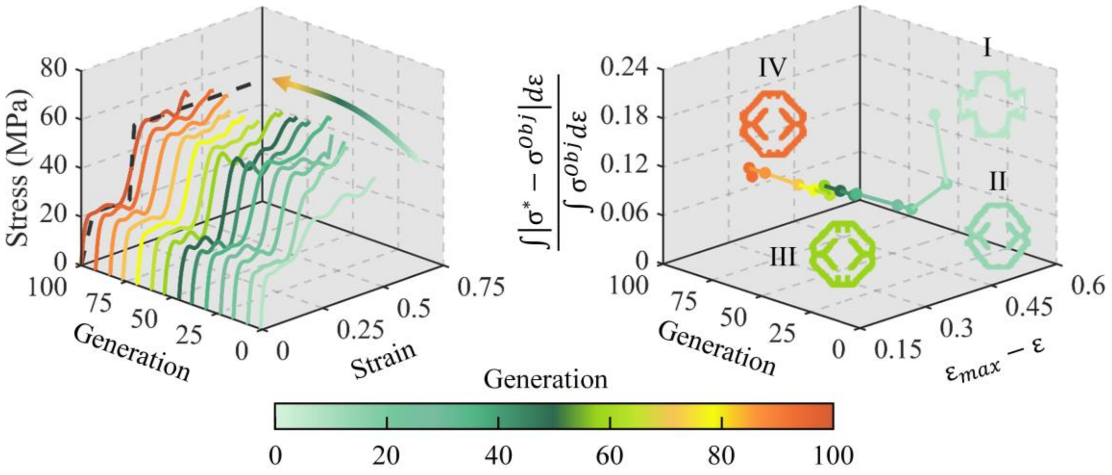

Figure S14. The optimization process of optimal structure with double platform stress.

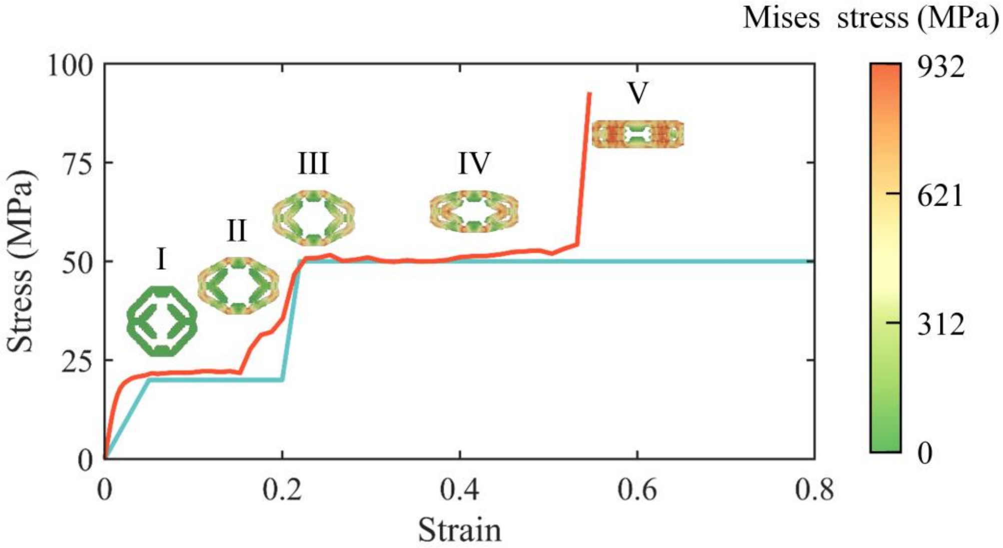

Figure S15. Deformation process of double platform energy absorption structure where I, II, III and IV represent the deformation state of the structure under a strain of 0, 0.18, 0.2, 0.4 and 0.6, respectively.

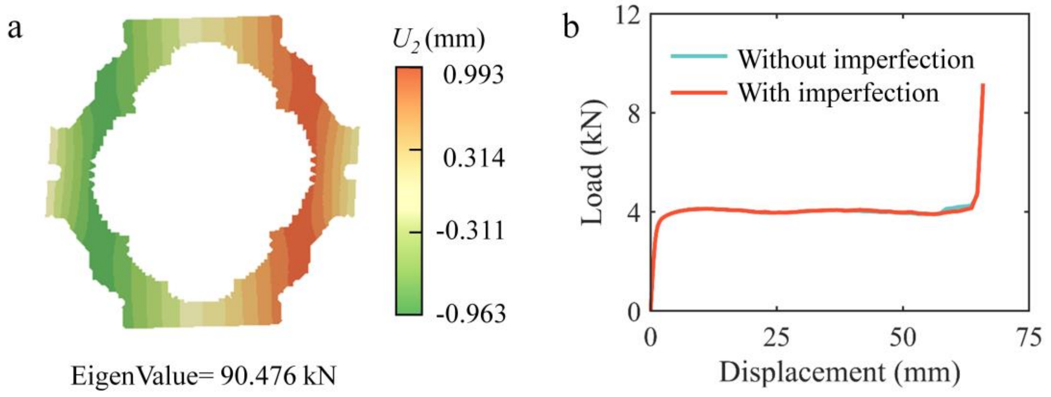

Figure S16 Buckling analysis of unit cells. a) First mode of structure with σ_m = 40MPa. b) The compression simulation results of model with imperfection and without imperfection of σ_m = 40MPa.

Table S1. The printing parameters of the test specimen.

Powder diameter [µm]	Powder layer thickness [µm]	Scanning power [W]	Scanning speed [mm/s]	Scanning pitch [mm]
17~53	40	250 W	850 mm/s	0.11 mm

Table S2. Design errors of structure with the same platform stress.

	Curve 1	Curve 2	Curve 3	Curve 4
S-unit	7.33%	7.12%	6.61%	17.30%
ε_m	0.5732	0.6478	0.5871	0.6885

Table S3. Simulation and experimental errors of an ideal energy-absorbing structure.

	σ_m = 10 [MPa]	σ_m = 20 [MPa]	σ_m = 40 [MPa]	σ_m = 60 [MPa]	σ_m=140 [MPa]	σ_m = 250 [MPa]
S-multi unit	2.80%	2.71%	4.02%	4.34%	5.35%	3.07%
S-multi error	7.63%	9.44%	10.37%	10.1%		
E-multi error	18.06%	13.84%	12.80%	19.11%		
ε_m	0.6628	0.6622	0.659	0.663	0.6794	0.5033
